# With advancement in health technology comes great responsibility – Ethical and safety considerations for using digital health technology: A narrative review

**DOI:** 10.1097/MD.0000000000039136

**Published:** 2024-08-16

**Authors:** Liza Grosman-Rimon, Pete Wegier

**Affiliations:** a Levinsky-Wingate Academic College, Wingate Institute, Netanya, Israel; b Research Institute, Humber River Health, Toronto, ON, Canada; c Institute of Health Policy, Management and Evaluation, University of Toronto, Toronto, ON, Canada.

**Keywords:** ethics, health technology, safety

## Abstract

The accelerated adoption of digital health technologies in the last decades has raised important ethical and safety concerns. Despite the potency and usefulness of digital health technologies, addressing safety, and ethical considerations needs to take greater prominence. This review paper focuses on ethical and safety facets, including health technology-related risks, users’ safety and well-being risks, security and privacy concerns, and risks to transparency and diminished accountability associated with the utilization of digital health technologies. In order to maximize the potential of health technology benefits, awareness of safety risks, and ethical concerns should be increased, and the use of appropriate strategies and measures should be considered.

## 1. Introduction

In the last few decades, the accelerated adoption of digital health technologies has raised important ethical and safety concerns.^[1]^ To date, the use of digital health technology tools by users such as patients, healthcare providers, and healthcare organizations is widespread, with the goal of improving health outcomes and delivery of care by providing informed treatment recommendations, clarifying and refining diagnoses, optimizing workflows and efficiency, improving self-care, and facilitating access to and use of complex healthcare data.^[2–4]^ Health technology is also used for health promotion, chronic disease prevention, and wellness.^[5,6]^

**Figure 1.**
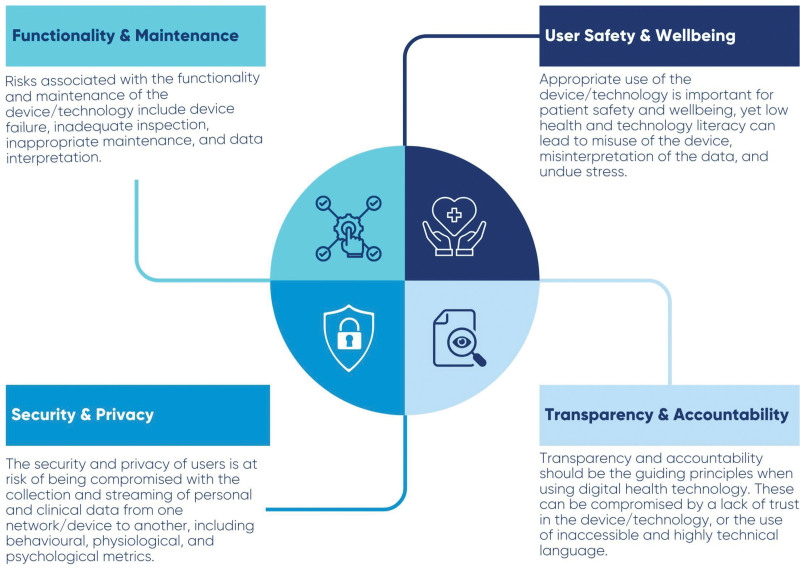
A description of safety risks and ethical concerns associated with digital health technology.

Importantly, health technology can also empower patients and technology users in their pursuit of health and wellness, allowing users to process and visualize their generated data. Despite the technology’s potency and usefulness, “with power comes responsibility” to ensure that safety and ethical considerations are in place.^[7]^ This review paper focuses on ethical and safety facets, including health technology-related risks, users’ safety and well-being risks, security and privacy concerns, and risks to transparency and diminished accountability associated with technological utilization (Fig. 1). Considerations and measures to ensure that ethical and safety concerns are addressed are also discussed.

## 2. Health technology-related risks

Although health technology is widespread in the healthcare setting, there are risks associated with its use related to the device/technology itself, its operation, and data interpretation and management. These risks include device failure, insufficient user training, inappropriate use, inadequate inspection, and inappropriate maintenance.^[8]^ These can lead to an inability to appropriately perform medical examinations, obtain important medical information, and monitor patients. Additional risk may be related to setting and infrastructure, including poorly trained service providers, limited resources and supervision, and inadequate electrical backup.^[8]^ There are also health risks due to data corruption or data loss due to power outages, power disruptions, improper shutdowns, hardware failure and dysfunction, or issues with programming. Another ethical issue is that patients who have no affordable or reliable access to internet or mobile technologies, who are marginalized,^[3]^ such as people in rural or isolated areas, may not receive optimal health services.

In a clinical setting, inaccurate measurements or unreliable devices may result in useless data, or the worst case, lead to harmful clinical outcomes.^[7]^ Thus, data used to predict risk or alert clinical staff may create false positives or false negatives.^[9]^ In addition, big data generated by different health technologies are massive in size, heterogeneous, and of differing quality, requiring sophisticated automated techniques to find clinical meaning and drive clinical insight.^[10]^ Several large health organizations have overcome some of these challenges by implementing clinical command centers.^[11–14]^ A command center uses algorithms and predictive analytics to integrate real-time data from multiple automated systems, supporting early identification of patients at risk of harm and deterioration.^[12]^ However, only a few health organizations around the world have implemented command centers.^[11–15]^

Indeed, many medical apps, health-promoting and wellness tools, and wearable devices are not formally categorized as “medical devices” and therefore are often not regulated.^[3,16,17]^ Other potential risks to patients with the use of digital data are due to self-diagnosis and self-treatment.^[10]^ The risks for lay people self-diagnosing using invalidated data with poor quality may be also related to the possibility that patients will not seek further advice from medical professionals and if they do, they may not adhere to the treatment.

The effects of these unregulated technologies on doctor-patient encountered, patients’ behavior, as well as clinical decision-making and practices are not fully understood and therefore a source for concern.^[16]^ It is not clear how healthcare providers are responding to self-diagnoses by their patients using unregulated devices and how providers then negotiate diagnoses and treatment options with their patients.^[16]^ There may be a possibility that the data obtained from unregulated health technology hinders the provision of optimal diagnoses and treatments. This may lead to overdiagnosis and overtreatment, which may result in a higher likelihood of overprescription of medications, iatrogenic complications, and suboptimal allocation of resources, so that patients who truly need care must wait longer.

On the other hand, appropriate education and training of healthcare providers is highly important. Healthcare providers should understand the meaning of the information provided by technology, how the technology works and how they should respond. Implementing an early warning system to identify patients who are at high risk of mortality, and who may benefit from a palliative approach to their care has been shown to appropriately identify those patients.^[18,19]^ However, additional work revealed that some healthcare providers felt that they did not receive enough information about how the score was calculated, were concerned about redundancy of the alert, experienced alert fatigue, or were unclear about the appropriate next step.^[20]^

Another concerning issue that may impact health and well-being is the possibility of patients becoming over-reliant on automated systems, providing a false sense of security.^[21]^ Passive monitoring may also pose a risk to patients,^[10]^ as patients may assume they are actively monitored by the healthcare providers, when in fact they are only monitored by the technology and not by health providers, which may only provide limited clinical benefits. In health organizations with command centers, it is common to rely on co-location of key clinical and operational staff, pooling collective skills and expertise to constantly monitor and respond to risk and deterioration of patients before they exacerbate. However, in many cases, patients are not constantly monitored.^[15]^

### 2.1. Considerations and measures to reduce technology-related risks

- Improving reliability and validity of the technological methods, in order to improve the consistency and accuracy of the data.
- Using algorithms and predictive analytics to integrate real-time data from multiple automated systems.^[12,15]^
- Implementing a co-location team design with collective skills and expertise that constantly monitors patients.^[15]^
- Implementing early warning systems for device failure.^[8]^
- Ensuring training of healthcare providers and medical technicians; establishing accreditation process and encouraging training and continued professional development; improving audit.
- Ensuring timely and inappropriate inspection and maintenance of devices.
- Ensuring seamless access to internet, especially in patients who are remotely being monitored.
- Instructing patients to use the telephone, in the event of internet interruption.
- Establishing the mechanisms of reporting adverse events and learning to identify areas where technology is unsafe.^[8]^
- Increasing awareness of the potentially harmful content of unregulated technology.^[17]^

## 3. Health technology users’ safety and well-being risks

Despite the benefits of health technology, there are several important concerns related to the patients using the technology that should not be ignored. Appropriate use of technology is important for patient safety and well-being. This can be achieved by educating patients and providing them with opportunities to enhance their technology literacy. In a study conducted on community patients with palliative care needs, a large proportion of patients used a remote symptom self-monitoring app to complete self-reporting assessments. Comprehensive training was provided to the patients prior to leaving the clinic on how to use the technology, including a “teach back” approach and an instructional booklet.^[22]^ However, it is possible that in many cases patients do not receive adequate training.

Furthermore, comprehensive education of patients is important to ensure adequate understanding of the process and procedures, as well as the capacity to interpret the clinical data generated by technology. Lack of adequate education may compromise therapeutic relationships, patient safety, and appropriate allocation of scarce healthcare resources.^[3]^ Patients’ understanding of the meaning of the data is highly important so that they adhere to their medical plan and seek help when needed. In addition, confusion and misuse of the technologies by the technology users may arise as a result of blurred boundaries between unregulated and regulated technology and different technological approaches related to healthcare delivery, self-care, self-initiated health promotion, preventive medicine, health education, communication, and these areas intersecting with one another.^[23]^ Patients should be educated to view data critically and to discriminate information based on quality.

Furthermore, appropriate use of technology requires a certain level of technology literacy. Disadvantaged social groups characterized by lower levels of income and education, poor language proficiency, and living in marginalized geographical locations may have low technology literacy.^[24–28]^ Also, elderly patients with inadequate technology literacy, who feel uncomfortable with technologies may be marginalized.^[3]^ Importantly, poor health outcomes may be exacerbated rather than improved if technology literacy is low and is used inappropriately and uncritically.^[23]^ Some patients do not have internet access, desktops, laptops, smartphones, or tablets, and probably less likely to feel comfortable using these technologies. In addition to the patients’ inadequate technology literacy, healthcare providers may also have trouble keeping up with technological developments, making it challenging for them to educate or warn patients accordingly.^[3]^

Another safety concern is addictive behavior, due to the design of health technology to be engaging for patients, by activating a reward system in the brain.^[29]^ For example, smartphone applications and internet abuse are associated with a range of psychological disorders, including sleep disturbance, anxiety, stress, and depression.^[30,31]^ Potentially, these psychological disorders can be similar with the use of many health technologies. Patients’ exposure to an abundance of health information, including test results, diagnosis, and prognosis, may be overwhelmed and stressed, affecting their well-being. Excessive self-monitoring may be uncomfortable, unpleasant and intrusive for patients who can also suffer from negative consequences.^[21]^ A study conducted on patients with newly diagnosed diabetes, reported that self-monitoring of blood glucose concentration had no effect on glycaemic control, but rather was associated with depression.^[32]^ In addition, an overabundance of constantly updated information about a patient’s health can be distracting and impede daily routine^[7]^ and productivity. For example, smartphone overuse and activated notifications are associated with low productivity and impede focus.^[33]^ In contrast, Gamified applications have the potential to improve patient self-management, medication adherence, and knowledge.^[34]^

### 3.1. Considerations and measures to reduce users’ safety and well-being risks

- Providing appropriate education and training for the use of health technology.
- Tailoring instructional martial to the level of the patients and providing opportunities to enhance technology literacy.^[32]^
- Using simple language and pictorial instructions for low-literacy users.
- Technology should be tailored to underserved patient populations (that are disadvantaged based on their ethnicity, age, gender, socioeconomic status, geographic locations, etc) to eliminate health disparities.^[35]^
- Monitoring patients’ well-being and emotional effects of technology used in addition to physiological outcomes.
- To mitigate compulsive, addictive, and distracted behaviors, technology could be programmed to initiate a ‘blackout when devices continue to track measurements during this period, while quantification functionality is unavailable for the patients.^[7]^

## 4. Security and data privacy concerns

One of the major consequences of health technologies’ is that the privacy and security of the technology users may not be well protected. Indeed, security and privacy risks can be related to the quantification of individual behaviors and physiological variables with a continuous stream of the data and its environments through the use of common networked devices and mobile sensing devices (e.g., smartphones, tablets, and wearable devices) and spatial location (using Global Positioning Systems [GPS], Bluetooth beacons, WIFI routers or cell phone towers).^[36,37]^ Concerns are raised about privacy and confidentiality breaches, regarding the collection and storage of highly detailed data such as individuals’ health, communication, media consumption, and spatial location.^[38]^ These data points may include time and date, altitude, latitude, longitude, and speed, providing detailed information about an individual’s whereabouts. In addition, sensing technology can estimate sleep, patterns, activity, mobility, physical movement, health-related habits, social engagement and behavior, communication, consumption, and mental health.^[23,38]^ Technologies involved in automated emotion recognition pose major concerns for patients, including body language and gesture recognition, facial recognition and facial expression analysis, speech recognition and speech emotion recognition, as well as pattern recognition, allowing the profiling of behavior and mental state. Ethical issues around data privacy and storage of photographs are also concerning, particularly when images and photographs contain sensitive data.^[39]^

Another concern is data linkage, a process of combining multiple datasets to increase the probability of user identification, due to the possibility that anonymized data may be linked to identifiable data or unique variables.^[38]^ Thus, there is a risk that anonymization of data via a distortion or removal of identifier does not always provide adequate levels of anonymity and is not sufficient enough to prevent identity fraud, since sophisticated algorithms and technological tools can cross-reference data with other “digital traces” of the users.^[21,40]^ Also, text messages, emails, and location data information – collected for non-explicit health care contexts – may pose risks to identifiability, which users may be unaware of.^[9]^ Activities that were once private or shared with the few now leave trails of data that expose the interests, traits, beliefs, and intentions of the many.^[41]^ The leakage of these sensitive data generated by health technology is a major concern. Therefore, data protection is especially critical because of the sensitivity of behavioral and health diagnoses information and their potential to have a significant impact on employment, insurance, litigation, or other aspects of life.^[9]^

In recent years, health centers have experienced an increase in cyberthreats that can have serious effects on patient safety. For example, cyber-attacks on a health technology may pose a threat to the safety of patients by controlling medication-delivering devices to change dosages to harm individuals.^[7]^ Security breaches of devices such as insulin pumps and pacemakers that have life-sustaining functions carry a major increased risk to the patients.^[42]^ Health technology devices can also be hacked maliciously with the intent to perform tasks like audio and video recording, opening web pages, delivering malware, deleting files, and being enslaved into botnets.^[7]^ To minimize the risk of compromising the confidentiality of personal information by cyber-attacks or hacking, data should be protected and technical measures should be in place to mitigate unwanted access to the data. In response, when implementing health technology, security measures need to be employed and strategies should be developed to ensure the safety of patients and technology users.

Misuse of data or third-party use of the data is also a major concern. Digital health technologies allow for the surveillance of individuals by organizations and the ability to collect and analyze data surreptitiously.^[9]^ Specifically, technology enables tracking patients’ data that may affect many aspects of patients’ live including credit, law enforcement, higher education, and pricing.^[10]^ There is potential to use digital traces to identify characteristics of an individual and abuse of the collected data by reusing or selling it to a third-party for commercial use,^[43]^ using these data in combination with other data or creating models to predict personality, financial situation, and credit scoring. Another example of misuse of data is that captured sensitive data by employers in health incentive programs.^[7]^ In cases where employees wear devices as part of workplace wellness programs, in which employers can view their personal and biometric data, and where there is an increased risk that encouragement becomes coercion, employees must give their consent.^[23]^

The concern of privacy and security breaches has led to legal regulation. The General Data Protection Regulation (GDPR) in the European Union (EU) regulates aspects regarding personal data and data management, which is based on a fine balance approach between protection and free flow of data.^[37]^ Similarly, The Health Insurance Portability and Accountability Act (HIPAA) is a United State (US) federal law that governs the privacy and security of personal health information as well as The Health Information Technology for Economic and Clinical Health (HITECH) Act which expanded the scope of privacy and security protections of HIPPA.^[44]^

### 4.1. Considerations/measures to protect privacy and confidentiality

- Institutions using the data would need to meet relevant data security requirements, with adequate data security measures in place to protect data and determine who can monitor the security of the data.^[9]^
- Data management decisions should be made. For example, determining which institutions, individuals, or users should store or have access to the raw data, the analytic system, and the reports generated by the system.^[9]^
- Manufactures of health technologies should anticipate future security threats and develop security features to address threats.^[7]^
- Minimizing the risk of compromising the confidentiality of personal information by providing patients with the ability to pause data recording before engaging in a private activity or delete data they do not wish to be recorded after they have been collected.
- When collecting identifiable data, clinicians and researchers should strive to anonymize the data and store de-identified data. Researchers should not link mobile sensing data with identifying information. In cases where it is not possible, data should be anonymized as soon as possible.^[38]^
- To protect users against unwanted intrusions into their personal data, guidelines should be developed to establish what kind of data may be gathered for certain types of uses.
- Password-protected devices should be stored in a safe protected space.
- Educating and raising awareness of security concerns and empowering patients to take ownership of the security of their devices, such as creating and using strong passwords are important.
- Providing technology end-users the option to “time-off” when data is not collected (turning off devices or certain functions, including camera, microphones and locations).

## 5. Risk to transparency and diminished accountability

Transparency and accountability should be the guiding principles when using digital health technology, especially in the context of safety and ethics. To achieve transparency, it is important to clarify what type of data is collected by digital technology, how it is collected, and when it is collected. Transparency also includes a description of how the technology works, how the data is collected and analyzed, and the potential limitations of the analysis tool. This may often constitute a challenge because the algorithms developed by the private companies are protected as intellectual property, they are not fully transparent, and as in the case of applying artificial intelligence, the algorithms constantly change as the system learns.^[9]^

In clinical research, transparency is important, especially when digital technology is utilized, as it allows patients to make informed choices and improves trust among researchers, healthcare providers, and patients. Importantly, transparency also facilitates the building of trust of digital technology in patients. However, results of implementation research have shown technologies that appear to be appealing, engaging, and effective in research settings often vary from what was reported in trials, and may not be efficacious in the real world.^[45,46]^ In many cases, the usability of technology in clinical settings to support patient management is not yet fully understood and needs to be thoroughly examined. In addition, transparency also includes how and when patients and research participants will be informed of digital phenotyping analyses, which may include personal data such as location, sleep cycle, or recordings of voice and speech, data that many people may be reluctant to share.^[9]^ Unfortunately, in many cases transparency is not achieved. Lack of transparency characterizes many aspects of information technologies used in healthcare or that involve health-related data, such as algorithms, telemedicine, EHR systems, mHealth, wearables, devices, and social networks.^[47]^ For example, research on privacy practices of a popular application for depression and smoking cessation revealed that information on data handling and data sharing with third parties that includes linkable identifiers are not being fully disclosed.^[48]^

One of the challenges when employing health technology is that human-led data input is prone to errors. In the event of an error, it should be made clear who will be accountable for the errors (e.g., clinicians, institutions, manufacturers), as well as the duties and obligations of different institutions and individuals involved in developing and implementing the technology should be determined and communicated clearly.^[9]^ Moreover, transparency also involves an end-user agreement, service terms, or a consent form, which is a document of rights and ethical and legal obligations. In the medical setting, the process includes disclosing to the patients the relevant information, as well as providing them with full information about the risks, benefits, and alternatives of using the health technology. The patients must have a sufficient level of comprehension to make voluntary and informed decisions about their participation and/or their decision to terminate the process. The patients should be informed and understand: what type of data and when their data are collected and by whom; where their data will be stored; who will have access to these data, and how their data could be used; what types of inferences will be made from the data and what is magnitude of likelihood error.^[9]^

Indeed, in the context of health technology, using digital tools for informed consent in clinical research was not found to negatively affect the process, but rather potentially enhance efficacy.^[49]^ The technology allows for dynamic interaction between researchers, clinicians, and patients during this consent process using media options, such as images, links, and videos, as well as engaging games and quizzes to test patients’ knowledge and convey important information.^[7]^ Indeed, interactive consent using digital technology was found to be valuable.^[50]^ Leveraging technological tools to transform the informed consent process into an interactive process rather than reading a paper format has a greater potential to foster an understanding of the research process and allows participants engagement.

### 5.1. Considerations/measures to achieve transparency and accountability

- Developers of digital technology tools and institutions using the technology will need to communicate how the technology works, the data that are being collected, as well as potential limitations.^[9]^
- Determining requirements for reporting failures of digital technology and data collection: what should be reported, to whom, and who should have access to the resulting information.
- Researchers and clinicians will need to describe precisely to patients and research participants what data they will and will not collect.
- Transparent communication by the researchers and clinicians regarding when and how patients and research participants will be informed of digital phenotyping analyses of their data for specific uses.^[9]^
- Transparent communication by the researchers and clinicians regarding data analysis, including the effectiveness, and limitations of the analysis and how digital data are translated into clinical insights.^[9]^
- The process should be transparent to the participants, informing them what the data might reveal about them, for how long the data will be used, who will be using it, and why.^[51]^
- Participants should be made aware and approve of all the data that is being collected and stored.
- The informed consent should include information about security requirements and measures in place to protect the data and who can monitor the security of the data.^[9]^
- Data management tools should be designed to help people manage their data, including the ability to define acceptable use, limit data access, delete data, or withdraw consent altogether.^[51]^

## 6. Conclusion

The immense numbers of digital health technology innovations used for health and wellness have tremendous potential to improve many aspects of health and health provision. However, many ethical and safety concerns should be acknowledged and addressed. While some of the risks are related to the technology and technologically derived data, other risks are related to the users and how technology is used. Digital health technology also raises risks to the security and privacy of patients. Some of these complex safety challenges and ethical issues if they are not addressed lead to a lack of transparency and accountability. Although safety is often part of the agenda when assessing technology, making decisions about policy, and adopting new technology,^[52–54]^ ethical issues are not often considered.^[55]^ To maximize the potential for health technology benefits, awareness of safety risks and ethical concerns should be increased, and the use of appropriate strategies and measures should be considered. Healthcare organizations should incorporate education to raise awareness of concerns with using digital health technology ethically and safely. Guidelines and policies, in addition to sharing experiences and exchanging information within and outside health organizations, are important for developing a culture of safety and ethics around the use of digital health technology.

## Acknowledgments

We wish to thank Kirsten L Ellison for preparing Figure 1.

## Author contributions

**Conceptualization:** Liza Grosman-Rimon.

**Validation:** Liza Grosman-Rimon.

**Writing – original draft:** Liza Grosman-Rimon.

**Writing – review & editing:** Liza Grosman-Rimon, Pete Wegier.

**Supervision:** Pete Wegier.
